# Theories, models and frameworks used in capacity building interventions relevant to public health: a systematic review

**DOI:** 10.1186/s12889-017-4919-y

**Published:** 2017-11-28

**Authors:** Kim Bergeron, Samiya Abdi, Kara DeCorby, Gloria Mensah, Benjamin Rempel, Heather Manson

**Affiliations:** 1 Public Health Ontario, 480 University Avenue, Suite 300, Toronto, ON M5G 1V2 Canada; 2 School of Public Health and Health Systems, University of Waterloo, 200 University Avenue West, Waterloo, ON N2L 3G1 Canada; 3 Dalla Lana School of Public Health, University of Toronto, 155 College Street, 6th floor, Toronto, ON M5T 3M7 Canada

**Keywords:** Capacity building, Public health, Theories, Models, Frameworks, Health promotion

## Abstract

**Background:**

There is limited research on capacity building interventions that include theoretical foundations. The purpose of this systematic review is to identify underlying theories, models and frameworks used to support capacity building interventions relevant to public health practice. The aim is to inform and improve capacity building practices and services offered by public health organizations.

**Methods:**

Four search strategies were used: 1) electronic database searching; 2) reference lists of included papers; 3) key informant consultation; and 4) grey literature searching. Inclusion and exclusion criteria are outlined with included papers focusing on capacity building, learning plans, professional development plans in combination with tools, resources, processes, procedures, steps, model, framework, guideline, described in a public health or healthcare setting, or non-government, government, or community organizations as they relate to healthcare, and explicitly or implicitly mention a theory, model and/or framework that grounds the type of capacity building approach developed. Quality assessment were performed on all included articles. Data analysis included a process for synthesizing, analyzing and presenting descriptive summaries, categorizing theoretical foundations according to which theory, model and/or framework was used and whether or not the theory, model or framework was implied or explicitly identified.

**Results:**

Nineteen articles were included in this review. A total of 28 theories, models and frameworks were identified. Of this number, two theories (Diffusion of Innovations and Transformational Learning), two models (Ecological and Interactive Systems Framework for Dissemination and Implementation) and one framework (Bloom’s Taxonomy of Learning) were identified as the most frequently cited.

**Conclusions:**

This review identifies specific theories, models and frameworks to support capacity building interventions relevant to public health organizations. It provides public health practitioners with a menu of potentially usable theories, models and frameworks to support capacity building efforts. The findings also support the need for the use of theories, models or frameworks to be intentional, explicitly identified, referenced and for it to be clearly outlined how they were applied to the capacity building intervention.

**Electronic supplementary material:**

The online version of this article (10.1186/s12889-017-4919-y) contains supplementary material, which is available to authorized users.

## Background

Public health practitioners engage in learning opportunities to build capacity, improve performance and enhance the quality of working environments in order to advance public health goals [1]. Capacity building is necessary to support effective health promotion practice [2] and is a required action in the Bangkok Charter for Health Promotion [3]. The World Health Organization defines capacity building as “the development of knowledge, skills, commitment, structures, systems, and leadership to enable effective health promotion” [3]. It involves actions to improve health at three levels: the advancement of knowledge and skills among practitioners; the expansion of support and infrastructure for health promotion in organizations, and; the development of cohesive partnerships for health in communities [4]. Therefore, in addition to focusing on developing individual and organizational capacity, capacity building consists of acquiring and applying new or enhanced capabilities to promote health and engage in evidence-informed interventions [5]. The aim of capacity building is to improve practices and infrastructure by creating new approaches, structures or values which sustain and enhance the abilities of practitioners and their organizations to address local health issues [5]. It also involves engaging in a series of relationships with others within and outside of an organization to build public health knowledge and skills [6]. Examples of organizations providing these types of capacity building services include the World Health Organization [7] at an international level, the Public Health Agency of Canada [8] and the Centre for Disease Control and Prevention [9] at national levels and Public Health Ontario at a provincial level [10].

Capacity building organizations typically provide services such as consultations, technical assistance, web-based learning options, relevant knowledge products and resources, and facilitated training sessions [11]. These types of organizations engage in a purposeful process with those seeking to increase their capacity in order to achieve a particular goal [12]. This process is called a capacity building intervention. Using theories, models or frameworks as a foundation for capacity building interventions can provide a road map for studying programs, developing appropriate interventions and evaluating their effectiveness [13]. They can also inform implementation practices and can highlight the interplay between actions and outcomes [14]. However, there is limited research around how best to design capacity building interventions to optimize effectiveness, with some academics arguing it is not always clear how concepts are applied or what theoretical foundation interventions are based upon [11].

The purpose of this systematic review is to identify underlying theories, models and frameworks used to support capacity building interventions relevant to public health practice. The aim is to inform and improve capacity building practices and services offered by public health organizations.

## Methods

The authors worked together to scope the purpose of this review and to construct a plan for implementation. This plan included: identifying a search strategy, determining inclusion and exclusion criteria, setting a process for screening papers, determining appropriate methodological quality assessment for studies, identifying potential data extraction headings and identifying strategies for synthesizing results.

### Search strategy

Four search strategies were used: 1) electronic database searching; 2) reference lists of included papers; 3) key informant consultation; and 4) grey literature searching. A systematic electronic database search was initially conducted by Public Health Ontario Library Services on September 29, 2015 and updated on September 29, 2016 in four databases: 1) Ovid MEDLINE, 2) Embase, 3) CINAHL Plus with Full Text, and 4) PsycINFO. The search aimed to locate capacity building articles in public health and general healthcare and included “Capacity building” [MeSH] as well as keywords related to theories (e.g., frameworks, models, steps, and/or specific types of theories) and capacity building approaches such as “competency-based education”, “technical assistance”, and/or “education”. See Additional file 1 for the full search strategy and terms used. We searched reference lists of included articles and conducted key informant consultations to identify additional references that might have been missed. Key informants included Public Health Ontario (PHO) Health Promotion Capacity Building team members [15] and managers of Ontario health promotion resource centres [16]. A grey literature search was conducted November 10, 2016 and included grey literature repositories, custom web search engines, and a general web search (see Additional file 2). Searches were limited to articles published in the last 11 years and in the English language.

### Inclusion and exclusion criteria

Articles were included if they were published in English over the last 11 years, were about capacity building, learning plans, professional development plans in combination with tools, resources, processes, procedures, steps, model, framework, guideline, described in a public health or healthcare setting, or non-government, government, or community organizations as they relate to healthcare, and must explicitly or implicitly mention a theory, model and/or framework that grounds the type of capacity building approach they have developed.

Exclusion criteria included non-English language papers published earlier than 2005, settings unrelated to healthcare, capacity building in developing and low resource countries, curriculum development in academic settings (e.g., university research centres and departments) and where there was no theory implied or explicitly stated.

### Screening and selection of studies

#### Electronic database

Titles and abstracts of all identified articles in the original 2015 search were screened by two review authors (KB and KD), who independently screened 20% of the search results for relevance and had an agreement score greater than 80%. The remaining 80% of results were split in half and independently screened (KB and KD). Two team members (KB and SA) independently screened the full set of the updated search conducted in 2016. At full-text relevance screening, two authors (KB and SA) independently screened 50%. In addition, each author reviewed 20% of the other authors’ full-text articles. Any discrepancies were resolved by discussion until consensus was reached. The reference lists of all relevant articles were screened to identify additional articles. Those additional papers were retrieved and screened for inclusion.

### Grey literature and key informant consultation

Titles and abstracts of all grey literature search results were screened by one author (KD). Full-text assessment of grey literature and all sources identified through key informant consultation were screened for relevance by two authors (KB and SA). Consensus was reached on all discrepanciesvia discussion between the two authors.

### Quality assessment

A quality assessment was performed to assess the methodological quality of included articles. Using Caldwell et al. [17] and Creswell [18] for guidance, a quality assessment tool was developed that included these six questions:

1. Is the methodology identified and justified?
2. Was a theoretical lens or perspective used to guide the study, with a reference provided?
3. Is the theoretical framework described?
4. Is the theoretical framework easily linked with the problem?
5. If a conceptual framework is used, are the concepts adequately defined?
6. Are the relationships among concepts clearly identified?

A scoring of yes, somewhat or no could be applied. Typically, a scoring of somewhat meant that some information was provided but not enough to score yes. Those questions that scored yes were added together for a final score. Articles that scored three or fewer yes ratings were classified as moderate and articles that scored four to six were classified as strong. Methodological quality was independently assessed by two authors (KB and SA). There were no disagreements on individual rating scores. Quality appraisal results for included papers are shown in Additional file 3 and indicate the quality score that resulted in moderate and strong ratings.

### Data extraction

A data extraction table was drafted and refined by discussion among the authors. Two authors (KB and SA) independently extracted data from five papers and met to discuss results. The resulting discussion generated a guide for data extraction by the two authors (KB and SA) to achieve consistency. Each author performed data extraction on a sub-set of included papers. In addition, each author reviewed 20% of the other authors’ data extraction and added any missing information. Information extracted from each paper included: author and year, purpose/objective, study design, intervention description, country and/or location/setting, organization and type of profession, context, theories and frameworks cited, theories and frameworks applied, findings/results, implications for practice, conclusion and study limitations.

### Data analysis

For the purpose of this paper, a systematic review is defined as an evidence synthesis that adheres to guidelines on the conduct of the review [19]. The Cochrane Health Promotion and Public Health Field Guidelines [20] were used to inform the process for synthesis and analysis of the articles, particularly the section on theoretical frameworks. The first six sections of the data extraction table which pertain to characteristics of the included papers were analyzed and presented as descriptive summaries.

The theories, models and/or frameworks cited were categorized according to which theory, or model, or framework they represented, and whether its reference was implied or explicitly stated. The following definitions from Nilsen [21] were used to categorize each theory, model and/or framework:

- Theories include constructs or variables and predict the relationship between variables;
- Models are descriptive, simplification of a phenomenon and could include steps or phases; and
- Frameworks include concepts, constructs or categories and identify the relationship between variables, but do not predict this relationship.

Once this was completed, each article was reread to identify whether the theory, model or framework was implied or explicitly identified. Articles were categories as implied if authors named the theory, model or framework but provided no additional information such as a reference and/or figure or description or if they did not name a theory, model or framework but did identify components. For example, if different levels such as individual, system, community or policy were presented, an ecological model approach [22] was implied and categorized as such. A theory, model or framework was categorized as explicitly stated if the authors stated the proper name and provided a reference to support the theory, model or framework identified. Lastly, based on the above analysis, articles that were categorized as explicit and included the most frequently cited theories, models and frameworks were reviewed to see how these theories, models or frameworks were used.

## Results

**Fig. 1:**
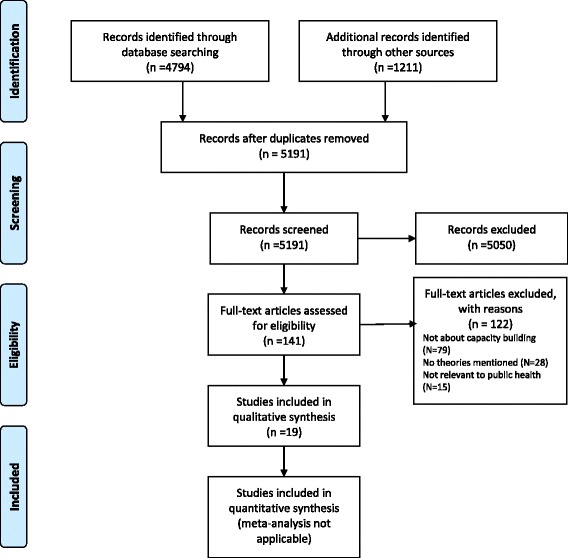
PRISMA flow diagram

The PRISMA flow diagram [23] reported in Fig. 1 depicts the process of selection and identification of articles. Our search strategy identified 5191 articles. Of these, 141 were selected for full-text review. Of the 141, 122 were excluded because they were not about capacity building, or no theories, models or frameworks were mentioned or they were not relevant to public health. As a result, 19 articles were included in this review.

### Summary of quality assessment

Eight papers [11, 24–30] were rated strong and 11 papers were rated moderate [31–41]. Most of the moderate ratings were due to theories, models or frameworks being implied versus explicitly stated. No papers were excluded due to quality.

### Overview of studies selected

Eleven papers were published between 2005 and 2011 [26, 28–30, 32, 33, 37–41] and eight published between 2012 and 2016 [11, 25, 27, 29, 31, 34–36]. Five of the papers were published in Canada [27, 28, 34, 35, 38], five in the United States [26, 30, 37, 40, 41], two in Australia [24, 33], two in Europe [29, 31] and one in the United Kingdom [39]. Four of the papers did not state a specific country [11, 25, 32, 36].

The purpose of the papers varied. For example, some papers focused on developing ‘systems’ capacity [26, 28, 31, 33–35, 38, 40], whereas other papers focused on developing field specific practitioner capacity (e.g., nutrition, mental health, pharmacists, infection disease control) [24, 27, 30, 32, 36, 37, 39]. Four papers focused on building individual capacity in areas such as technical assistance [25], evidence-based interventions [11], coaching [41], and policy [29]. Nine of the papers specifically focused on public health practice [26, 28, 30–34, 38, 41].

Related to study design, nine of the papers included a literature review as part of their methods [11, 24, 25, 27, 31, 32, 34–36], five were case studies [28, 29, 37, 39, 41], and three were commentaries [26, 38, 40]. One study [33] used in-depth interviews and workshops to explore date collected with public health experts and another study used a quasi-experimental design [30].

Table 1 provides an overview of the reference, country and purpose of the papers and identifies the underlying theories, models and/or frameworks, whether or not they were implied or explicitly stated and capacity building approaches found.

**Table 1:** Description of individual studies related to underlying theories, models and/or frameworks and capacity building approach

References	Country	Study method/design	Purpose	Underlying theory; explicit or implied	Underlying model; explicit or implied	Underlying frameworks; explicit or implied	Capacity building approach
Aluttis et al. [31]	Europe	Literature review Focus group	Review of currently existing frameworks, to highlight commonalities, and to propose a public health countrylevel framework which integrates all reoccurring dimensions into one framework	None	None	Capacity Mapping Framework- explicit	Concept mapping
Ballie et al. [32]	Not stated	Literature review	Describe a conceptual framework to assist in the application of capacity-building principles to public health nutrition practice	None	Ecological model- implied	None	Capacity building
Bagley and Lin [33]	Australia	Interviews Workshop	Develop and pilot test a rapid assessment tool to improve local public health system capacity	Diffusion of Innovations- implied	Ecological model- implied	None	Capacity mapping
Bamberg et al. [24]	Australia	Interviews Informal conversations Interviews	Present a project that utilized Donald’s Ely’s model to build research and evaluation capacity in a community health centre	Diffusion of Innovations-explicit	Ely’s Change Model-explicit	None	Capacity building
Cohen et al. [34]	Canada	Literature review Interviews Meeting	Present a Conceptual Framework of Organizational Capacity for Public Health Equity Action grounded in the experience of Canadian public health equity champions, that can be used as a tool to guide research, dialogue, reflection and action on public health capacity development to achieve health equity goals	None	Ecological model- implied	None	Capacity building
Hu, Rao and Sun [26]	United States	Commentary	Provide a scientific guide for most countries in the world to build a complete public health emergency management system	None	Capacity Assessment Theory- explicit Prevention, Preparation, Response, Recovery Model- explicit	None	Capacity assessment
Katz and Wadersman [25]	Not stated	Literature review	Focus on evidence based for technical assistance using three frames: tasks, relationships, and connections to the life cycle stage of an innovation (e.g., programs, policies, or practices that are new to a setting)	None	Interactive Systems Framework for Dissemination and Implementation - explicit Getting to outcomes- explicit	None	Technical assistance
Khenti et al. [35]	Canada	Literature review Focus group	Present a framework for global mental health capacity building that could potentially serve as a promising or best practice in the field	None	Developmental Evaluation Model- explicit	None	Capacity building
Leeman et al. [11]	Not stated	Literature review Interview Member checking	Conduct a scoping review to advance theory to guide the design of capacity building strategies, with a specific focus on strategies to adopt and implement community-based prevention evidence-based interventions	None	Interactive Systems Framework for Dissemination and Implementation - explicit	None	Capacity building
Meeker et al. [36]	Not stated	Literature review Interviews	Develop a technical competency framework for emergency nutrition preparedness, response, and recovery within the humanitarian system	None	Ecological model- implied	Bloom’s Taxonomy of Learning- explicit	Capacity building
Millery [37]	United States	Case study	Describe a capacity building program that extends the traditional mission of HIV/AIDs professional training programs	Diffusion of Innovations- implied	Community Leadership Development Model- explicit	None	Capacity building
Murphy et al. [27]	Canada	Literature review Pre-post survey	Design programs and interventions that capitalize on the knowledge, skills, and accessibility of pharmacists for improving mental health outcomes in communities	Behaviour Change Theoryexplicit	The Consolidated Framework for Implementation Research-explicit	Promoting Action on Research Implementation in Health Systemexplicit COM-B (C-capability, O-opportunity, M-motivation, and B- behaviour) Assessmentexplicit	Capacity building
Norman et al. [38]	Canada	Commentary	Describe a model that leverages and reconfigures social networks in a manner designed to support innovation within health systems	Social Learning Theories- implied Transformatio nal Learning Theory- implied Diffusion of Innovations- implied	Social Network Theory- implied	Complex Network Electronic Knowledge Translation Research Model- implied	Linking knowledge to action
Olley [39]	United Kingdom	Case study	Develop and implement a continuing education strategy within an infection prevention and control service	None	Lewin’s Freeze-Thaw Modelexplicit Seven S Modelexplicit Strengths, Weakness, Opportunities and Threats (SWOT) Analysis Model- explicit Political, economic, social and technological (PEST) Analysis Frameworkexplicit Feasibility Model- explicit	Action Learning Framework- explicit	Continuing education
Preskill and Boyle [40]	United States	Commentary	Offer a multidisciplinary model of evaluation capacity building	Diffusion of Innovationsimplied	Ecological model- implied	Bloom’s Taxonomy of Learning- implied	Technical assistance
Risley and Cooper [41]	United States	Case study	Describe the theory and methodology behind a program that incorporates individual and group coaching as a career and leadership development tool in both academic and applied public	Transformational Learning Theory- implied	Adult Learning Theoriesimplied Appreciative inquiry- explicit	None	Professional coaching
Robinson et al. [28]	Canada	Case study	Examine the utility of a linking system approach to support capacity building and dissemination of heart health promotion	Diffusion of Innovations- explicit	None	Linking Systems Approach- explicit	Linking knowledge to action
Rutten, Gelius and Abu-Omar [28]	Europe	Case study	Outline a theoretical framework for an interactive, research-driven approach to building policy capacities in health promotion	None	None	Analysis of Determinants of Policy Impact (ADEPT) Model- explicit	Linking knowledge to action
Stark et al. [30]	United States	Quasiexperimental	Assess the impact of an online continuing education course on knowledge, skills, and selfefficacy of nutrition professionals to use an ecological approach to prevent childhood obesity	None	Ecological model- explicit	None	Online continuing education

Description of included papers

### Underlying theories

Four individual theories were identified. The underlying theories cited most frequently as contributing to framework development were the Diffusion of Innovations (*N* = 6) [24, 28, 33, 37, 38, 40], followed by Transformational Learning Theory (*N* = 2) [38, 41], Social Learning Theories (*N* = 1) [38] and Behaviour Change Theory (*N* = 1) [27]. Of the theories identified, seven were implied; meaning that the names of the theories were provided but a specific reference to support the theory was not. Bamberg [24], Murphy [27] and Robinson [28] explicitly named the theory and provided an appropriate reference to support the theory named. This contributed to their quality rating as ‘strong’.

### Underlying models

Seventeen individual models were identified. The underlying models cited most frequently as contributing to framework development included the Ecological Model (*N* = 6) [30, 32–34, 36, 40], and Interactive Systems Framework (ISF) for Dissemination and Implementation (*N* = 2) [11, 25]. Although ISF has the word “framework” in its title, using Nilsen’s [21] definitions, we categorized it as a model as it included the attributes of a model (e.g., descriptive, includes three phases). The following models were each mentioned once: Ely’s Change Model [24], Capacity Assessment Theory and Prevention, Preparation, Response, Recovery Model [26], Getting to Outcomes [25], Developmental Evaluation Model [35], Community Leadership Development Model [37], The Consolidated Framework for Implementation Research [27], Social Network Theory [38], Lewin’s Freeze Thaw Model, Seven S Model, SWOT Analysis Model, PEST Analysis Framework and Feasibility Model [39] and Adult Learning Theories and Appreciative Inquiry [41]. Of the models identified, five papers implied an ecological model approach [32–34, 36, 40], one paper implied Social Learning Theory [38] and another implied Adult Learning Theories [41]. The other papers identified were explicitly cited and included an appropriate reference to support the model identified.

### Underlying frameworks

Seven individual frameworks were identified. Bloom’s Taxonomy of Learning was cited twice (*N* = 2) [36, 40]. The remaining frameworks were mentioned once: Capacity Mapping Framework [31], Promoting Action on Research Implementation in Health Systems and COM-B Assessment [27], Complex Network Electronic Knowledge Translation Research Model [38], Action Learning Framework [39], Linking Systems Approach [28], and Analysis of Determinants of Policy Impact (ADEPT) Model [29]. Of the frameworks identified, Bloom’s Taxonomy of Learning was implied once [40] and explicitly stated once [36]. Six frameworks were explicitly stated and one was implied (see Table 1).

### Capacity building approaches

When reviewing the papers to identify specific types of capacity building approaches, eight of the papers were found to focus on the overall concept of capacity building [11, 24, 27, 32, 34–37], three focused specifically on linking knowledge to action [28, 29, 38], two on capacity mapping [31, 33], two on technical assistance [25, 40] and two on continuing education [30, 39]. Capacity assessment [26], continuing education [39], and professional coaching [41] were each identified once.

Taken together, a total of 28 theories, models and frameworks were identified in this review. Of these 28, the most frequently cited theories were Diffusion of Innovations and Transformational Learning Theory. The models cited most often were the Ecological Model and Interactive Systems Framework for Dissemination and Implementation and the most frequently cited framework was Bloom’s Taxonomy of Learning. There was not one specific capacity building strategy identified most often. Capacity building approaches identified included training, technical assistance, knowledge networks, and professional coaching.

### Theories, models and frameworks

Two articles [24, 28] explicitly identified using the Diffusion of Innovations theory; however, only one of the articles [28] was assessed to have used it when developing capacity building approaches. Bamberg et al. [24] considered Ely’s Eight Conditions for Change model and Roger’s Diffusion of Innovations as relevant for their project and after comparing them, chose to use Ely’s eight conditions. Whereas, Robinson et al. [28] discussed how the concept of a linking systems approach to dissemination has its origins in Roger’s Diffusion of Innovations, specifically outlining an active two-way linking relationship between those who develop innovations (resource groups) and those who adopt them in practice (user groups). Using this concept, Robinson et al. [28] outlined a linking system approach to dissemination that is “aimed at supporting the transfer and uptake of public health innovations through 1) capacity building and 2) communication strategies to support evidence-based practice and program implementation.”

No articles were identified as explicitly stating how the Transformational Learning Theory was applied in capacity building approaches.

One article [30] was identified explicitly as applying an ecological model approach. Stark et al. [30] identified the use of an ecological approach to develop an online course to increase nutrition professionals’ knowledge, skills, and self-efficacy to prevent childhood obesity. The aim was that once trained “these local professionals can facilitate community-based collaborations to implement environmental interventions to support healthful eating and active living” [30]. The ecological approach was modeled in the course objectives. For example, objectives were included related to assessing and prioritizing individual behaviours, and environmental factors.

Two articles [11, 25] explicitly applied ISF. Katz and Wandersman [25] used it as a framework to inform their technical assistance approach. For example, the technical assistance provider works closely with the individuals and organizations that require assistance and determines the focus of capacity building interventions based on need. Leeman et al. [11] conducted a review that was guided by the ISF to advance theory to guide the design of capacity building interventions with a focus on strategies to adopt and implement community-and evidence-based interventions. This review framework “posits that CB [capacity building] strategies affect practitioners’ capacity, which in turn effects the extent and quality of delivery systems’ EBI [evidence-based interventions] adoption and implementation.” They concluded that “little is known about how to design CB [capacity building] strategies and even less about how best to tailor them to practitioners’ varying needs” [11].

One article [36] explicitly applied Bloom’s Taxonomy of Learning. Meeker et al. [36] constructed a competency framework that included core professional competencies (e.g., behaviours such as the ability to communicate and work effectively with others) and core humanitarian competencies (e.g., the application of humanitarian principles). The competencies identified were each assigned technical domains (e.g., advocacy, analytical skills, and leadership) and expressed in a form of behavioural indicators. These behavioural indicators were developed using “a revised version of assigned Bloom’s taxonomy of learning behaviour as a guide.”

In summary, of the five theories, models and frameworks most frequently cited, Diffusion of Innovations was used to outline a linking system approach of dissemination to support the transfer and uptake of innovations. An ecological model approach was used to develop learning objectives to assess and prioritize behaviours and factors at multi-levels such as individual and environmental. ISF was used to design capacity building strategies with a focus on the extent and quality of the delivery system, and Bloom’s Taxonomy of Learning was used to develop behaviour indicators.

## Discussion

This review can be viewed as a first step towards identifying specific theories, models and frameworks used to support capacity building efforts. Five underlying theories models and frameworks used to support capacity building interventions relevant to public health practices were identified. These findings can be used to better design capacity building interventions. For example, both Diffusion of Innovations and Transformational Learning are behavior change theories that can be used to guide the development of capacity building interventions [13]. At the design stage of an intervention, considerations such as the perceived attributes of the intervention, how decisions will be made, how the intervention will be communicated, what social structures and networks will be utilized and finally who will be promoting the intervention are all essential in encouraging uptake [42]. Adult and transformational learning theories are valuable for planning interventions that are intentional in shifting paradigms, expanding perspectives and allowing for self-reflection and autonomy. Applying these theories provides a guide to amplify the magnitude of the capacity building intervention adoption and sustainability [41, 42].

A unique contribution of this review is categorizing the theories, models or frameworks based on their attributes and not treating them as the same. This was done by utilizing definitions by Nilsen [21]. This exercise helped better explain how the underlying theories, models and frameworks could be used when designing a capacity building intervention for public health professionals. For example, Diffusion of Innovations and Transformational Learning as theories include variables and predict relationships between these variables whereas an ecological model approach and ISF include the description of phases or steps. This categorizing exercise brought to light that sometimes a name may include one of the three terms; however, this may not adequately describe their attributes. For example, ISF has the word “framework” in its title; however, using Nilsen’s [21] definitions, we categorized it as a model as it included the attributes of a model (descriptive, includes three phases). Based on this review, the lack of common definitions of theories, models or frameworks makes it difficult to determine how to apply them to an intervention designed to build capacity.

Another contribution of this review is the need to identify whether or not theories, models or frameworks within studies were implied or explicitly stated. Applying a theoretical foundation provides a systemic, logical pathway for an intervention to succeed. Therefore, capacity building practitioners are interested to understand, select and apply best-fit theories, models and/or frameworks to guide their design and implementation processes [21]. This review found that a limited number of published capacity building interventions identify a theoretical foundation. Interventions that did explicitly state a specific theory, model or framework, in most cases, did not explain how their concepts were applied. An implied theory, model or framework relies on the prior knowledge and interpretation of the reader which could be mistaken or biased. A clearly articulated and referenced theory, model or framework provides clarity on the conceptual footing of the intervention and helps to illustrate the relationship between various components of the intervention and the desired outcomes.

### Study limitations and strengths

These findings are limited in that there may be other relevant documents beyond published articles and grey literature searches, which are not available in the public domain. As a result, the listed theories, models and frameworks may not be exhaustive. Further, where the original researchers did not classify their approach as a theory, model or a framework the authors of this paper classified the approaches based on their understanding of the categories provided by Nilsen [21]. The authors of this paper identified implied theories in the literature based on their expertise in the public health field and knowledge of health promotion theories which may contain potential biases.

An applicable critical appraisal tool for this type of research was not readily available; therefore, a tool was developed by the lead author adapted from Caldwell et al. [17] and Creswell [18]. Assigning a score to each article and determining the strength of its quality might be biased based on the authors’ understanding of how a theory was applied in each article. Furthermore, this review was restricted to capacity building within public health and there may be other relevant literature in other fields such as knowledge exchange, implementation science, and community building which was not captured.

Strengths of this review include the authors’ collective experience working in a capacity building organization, the use of a comprehensive search strategies (e.g., four strategies were used) and assessing the quality of included articles.

### Implications for practice and research

This review provides public health practitioners with a menu of potentially useable theories, models and frameworks as a foundation to support capacity building program design and implementation. Our findings can be used to help guide implementation practice by encouraging practitioners to consider what underlying theories, models and/or frameworks could be used when designing capacity building interventions. Furthermore, our findings highlight the importance to explicitly identify and clearly define how theories, models and frameworks are used during various stages of the capacity building process. Lastly, this paper supports practitioners to consider that theories, models and frameworks have different attributes and to not treat them as being the same. For example, this review provides evidence of the importance of categorizing whether or not a capacity intervention includes a theory, model and/or framework and not grouping them all under the heading ‘theories’.

Further research could include conducting an environmental scan of public health capacity building organizations at the international, national and local level to identify their current use of theories, models and frameworks for capacity building interventions and comparing categorize the findings using the definitions provided by Nilsen [21]. This could include conducting a review of organizational policies and guidelines as well as conducting key informant interviews to discern when and how theories are applied to capacity building interventions, and if and how effectiveness is measured. The results could be compared to the findings of this paper to show alignment and/or differences.

### Conclusions

This review identifies specific theories, models and frameworks to support capacity building interventions relevant to public health organizations. The findings add a new lens to consider when designing capacity building interventions. Five theories, models and frameworks were identified for consideration as a theoretical foundation for designing and implementing capacity building approaches: 1) Diffusion of Innovation Theory; 2) Transformational Learning Theory; 3) Ecological Model; 4) Interactive Systems Framework for Dissemination and Implementation Model; and 5) Bloom’s Taxonomy of Learning Framework.

The findings support the need for the use of theories, models and/or frameworks to be intentional, explicitly identified, referenced and clearly explained, and for it to be clearly outlined how they were applied to capacity building interventions. Furthermore, this review underscores the need for capacity building practitioners to expand their knowledge and understanding of theories, models, and frameworks that are a best fit for capacity building interventions.

## Additional files

Additional file 1: Appendix A: Academic search strategy and terms used. (PDF 189 kb)

Additional file 2: Appendix B: Grey literature search strategy. (PDF 258 kb)

Additional file 3: Appendix C: Quality appraisal results for included papers. (PDF 207 kb)

## Abbreviations

CDC: Centers for Disease Control and Prevention
COM-B: capability opportunity motivation and behaviour
EBI: Evidence-based interventions
ISF: Interactive Systems Framework
NGO: Non-governmental organizations
PHAC: Public Health Agency of Canada
WHO: World Health Organization
