# Supplementary material for: Theories, models and frameworks used in capacity building interventions relevant to public health: a systematic review
Source: BMC Public Health. 2017 Nov 28;17:914. doi: 10.1186/s12889-017-4919-y (PMC5706342; doi:10.1186/s12889-017-4919-y)
Supplement: Supplementary file 1 — Appendix A: Academic search strategy and terms used. (PDF 189 kb) [file 12889_2017_4919_MOESM1_ESM.pdf]

Additional file 2 Appendix A: Search Terms

A systematic electronic search was conducted September 29, 2015 in Ovid MEDLINE (1946 to Present), Embase (1974 to 2015 Week 39), CINAHL Plus with Full Text, and PsycINFO (2002 to September Week 3 2015). An update was run September 29, 2016.

#	Searches	Results
**1**	Capacity Building/ or ("capacity building" or "prevention capacity" or (system* adj2 capacit*) or (("health promotion" or "public health") adj5 capacit*) or ((build* or increas* or develop* or enhanc* or strengthen*) adj5 (capacit* or skill* or abilit* or workforce)) or "learning plan*").ti,kw,kf.	8744
**2**	Diffusion of Innovation/ or Knowledge Management/ or Models, Educational/ or Models, Nursing/ or Models, Organizational/ or Models, Psychological/ or Models, Theoretical/ or Systems Theory/ or (theor* or framework* or construct? or model* or concept* or heuristic* or lens or paradigm* or principle? or pre-engagement or phase? or stage? or "innovation support").ti,kw,kf. or (theor* or framework* or construct? or model* or concept* or heuristic* or lens or paradigm* or principle? or pre-engagement or phase? or stage? or "innovation support").ab. /freq=2	2224244
**3**	Competency-Based Education/ or Computer-Assisted Instruction / or Consultants/ or Education, Distance/ or Education, Professional/ or Education/ or Educational Technology/ or Evidence-Based Medicine/ or Evidence-Based Nursing/ or Evidence-Based Practice/ or exp Education, Continuing/ or Government Programs/ or exp Health Personnel/education or Health Planning Technical Assistance/ or Inservice Training/ or Learning/ or Mentors/ or Planning Techniques/ or Preceptorship/ or Program Development/ or Program Evaluation/ or Staff Development/ or Teaching/ or ("best practice*" or "change agent*" or "learning plan*" or "promising practice*" or (evidence adj3 (base* or inform*)) or approach* or assist* or consultant* or educat* or effectiv* or elearn* or implement* or initiative* or instruct* or intervention* or learn* or mentor* or preceptor* or program* or strategies or strategy or support* or teach* or tool or tools or train* or webinar* or workshop* or "technical assistance").ti,kw,kf. or ("best practice*" or "change agent*" or "learning plan*" or "promising practice*" or (evidence adj3 (base* or inform*)) or approach* or assist* or consultant* or educat* or effectiv* or elearn* or implement* or initiative* or instruct* or intervention* or learn* or mentor* or preceptor* or program* or strategies or strategy or support* or teach* or tool or tools or train* or webinar* or workshop* or "technical assistance").ab. /freq=2	2964774
**4**	Health Occupations/ or Health Personnel/ or manpower.fs. or Personnel Delegation/ or Personnel Management/ or Personnel Selection/ or Personnel Turnover/ or Professional Autonomy/ or Professional Competence/ or Professional Practice/ or ("human resource*" or employee* or employer* or manpower or personnel or practitioner* or professional* or provider* or staff or worker* or workforce).ti,kw,kf.	331406
**5**	Community Health Planning/ or Health Care Reform/ or Health Planning Technical Assistance/ or Health Planning/ or Health Systems Agencies/ or Health Systems Plans/ or Organizational Case Studies/ or Organizational Culture/ or Organizational	845591

	Innovation/ or Organizational Objectives/ or Regional Health Planning/ or Systems Analysis/ or (system or systems or systemic or "health care" or "health administration" or (health adj3 (plan* or reform*)) or organization*).ti,kw,kf.	
**6**	Health Promotion/ or Public Health Administration/ or Public Health Practice/ or Public Health/ or ("public health" or "health promot*" or "health unit*" or "health authorit*" or "health department*" or "community health" or "population health").ti,kw,kf.	183438
**7**	1 and (2 or 3) and (4 or 5 or 6)	1887
**8**	limit 7 to english language	1839
**9**	limit 8 to last 10 years	1484
**10**	(exp Africa/ or exp Caribbean Region/ or exp Central America/ or exp Latin America/ or exp South America/ or exp Asia/ or Mexico/ or Developing Countries/) not (North America/ or exp Canada/ or exp United States/ or exp Australia/ or New Zealand/ or exp Europe/ or exp Developed Countries/)	845509
**11**	9 not 10	1238
**12**	remove duplicates from 11	1186
