# Supplementary material for: Theories, models and frameworks used in capacity building interventions relevant to public health: a systematic review
Source: BMC Public Health. 2017 Nov 28;17:914. doi: 10.1186/s12889-017-4919-y (PMC5706342; doi:10.1186/s12889-017-4919-y)
Supplement: Supplementary file 2 — Appendix B: Grey literature search strategy. (PDF 258 kb) [file 12889_2017_4919_MOESM2_ESM.pdf]

Additional file 2 Appendix B: Grey Literature Search Terms

Google https://www.google.ca/

**Search strategy**	**Date searched**	**# results retrieved**	**# results reviewed**	**# results selected**
"public health" OR "health promotion" capacity OR skill OR ability OR workforce build OR increase OR develop OR enhance OR strengthen	Nov 10,2016	154,000,000	50	7
"public health" OR "health promotion" capacity OR skill OR ability OR workforce build OR increase OR develop OR enhance OR strengthen	Nov 10,2016	154,000,000	50	1E
"public health" OR "health promotion" capacity building change-agent OR consultant OR workshop OR webinar	Nov 10,2016	2,800,000	50	2E
"public health" OR "health promotion" capacity building theory OR framework OR model OR concept OR heuristic OR paradigm OR principle OR stage OR lens	Nov 10,2016	3,720,000	50	4
"public health" OR "health promotion" capacity building learning OR teaching OR instruction OR education OR training OR mentor OR preceptor	Nov 10,2016	4,140,000	50	3
"public health" OR "health promotion" capacity building human-resources OR employee OR employer OR manpower OR health-personnel OR health practitioner OR health-professional OR health-provider OR staff OR workforce OR health-system	Nov 10,2016	39,800,000	50	0
"prevention capacity" OR "health promotion capacity" OR "public health capacity"	Nov 10,2016	126,000	50	0
