# Supplementary material for: Theories, models and frameworks used in capacity building interventions relevant to public health: a systematic review
Source: BMC Public Health. 2017 Nov 28;17:914. doi: 10.1186/s12889-017-4919-y (PMC5706342; doi:10.1186/s12889-017-4919-y)
Supplement: Supplementary file 3 — Appendix C: Quality appraisal results for included papers. (PDF 207 kb) [file 12889_2017_4919_MOESM3_ESM.pdf]

Additional file 3 Appendix C: Critical Appraisal

The development of this critical appraisal is adapted from Caldwell, Henshaw & Taylor (2011) and Creswell (2014). All 19 articles were assessed. All the 'yes' answers were added together for a final score. Articles that score three or below were classified as 'moderate' and articles that scored four to six were scored as 'strong'

**Questions/References**	**Altuttis**	**Ballie**	**Bagley**	**Bamberg**	**Cohen**	**Hu**	**Katz**	**Khenti**	**Leeman**	**Meeker**	**Millery**	**Murphy**
1. Is the methodology identified and justified?	yes	Yes; Literature Review (LR) conducted/ Only one method used	Yes; Interviews and workshop	Yes; LR interviews	Yes; LR Interviews (I)	n/a; No methods section commentar y	yes	yes	yes	yes	yes	yes
2. Was a theoretical lens or perspective used to guide the study?	Not clearly defined	Not explicitly stated EM implied	Not explicitly stated EM implied	yes	Not explicitly stated EM implied	yes	yes	yes	yes	Somewhat; Bloom's named EM implied	yes	yes
3. Is the theoretical framework described?	no	no	no	yes	no	yes	yes	Yes; however "relevant theories" was stated but not named	yes	Yes for Bloom's not for EM	somewhat	yes
4. Is the theoretical framework easily linked with the problem (or does it seem forced)?	n/a	n/a	n/a	yes	n/a	yes	yes	somewha t	yes	somewhat	Somewhat	yes

Questions/References	Altuttis	Ballie	Bagley	Bamberg	Cohen	Hu	Katz	Khenti	Leeman	Meeker	Millery	Murphy
5. If a conceptual framework is used, are the concepts adequately defined?	n/a	n/a	n/a	yes	n/a	yes	yes	somewha t	yes	somewhat	somewhat	yes
6. Are the relationships among the concepts clearly identified?	n/a	n/a	n/a	yes	n/a	yes	yes	somewha t	yes	Somewhat	somewhat	yes
Total number of 'yes'	1	1	1	6	1	5	6	3	6	2	2	6

Questions/References	Norman	Olley	Preskill and Boyle	Risley	Robinson	Rutten	Stark
1. Is the methodology identified and justified?	Somewhat describes the process used but not in great detail	Somewhat; used a survey method but the actual questions were not listed	No methods section; overview of the model; did say the model was developed on literature	Yes; even though there is no methods section;<br>Case study; no methods section	Yes; case study design; describes how the cases were choosen	Yes; case study	yes
2. Was a theoretical lens or perspective used to guide the study?	yes	yes	Somewhat<br>EM implied; Blooms and Diffusion of Innovation named but no references provided	Somewhat; no reference for Adult learning theories but a reference used for Appreciation Inquiry	yes	yes	yes
3. Is the theoretical framework	Somewhat; named but not referenced	yes there was a lot used	somewhat	No	yes	yes	yes

Questions/References	Norman	Olley	Preskill and Boyle	Risley	Robinson	Rutten	Stark
described?		References were provided for all.					
4. Is the theoretical framework easily linked with the problem (or does it seem forced)?	Somewhat	yes	somewhat	n/a	yes	yes	yes
5. If a conceptual framework is used, are the concepts adequately defined?	Somewhat	somewhat	somewhat	n/a	yes	yes	yes
6. Are the relationships among the concepts clearly identified?	Somewhat	somewhat	Somewhat	n/a	yes	yes	yes
**Total number of 'yes'**	1	3	0	1	6	6	6
